# Snail Promotes Epithelial Mesenchymal Transition in Breast Cancer Cells in Part via Activation of Nuclear ERK2

**DOI:** 10.1371/journal.pone.0104987

**Published:** 2014-08-14

**Authors:** Bethany N. Smith, Liza J. Burton, Veronica Henderson, Diandra D. Randle, Derrick J. Morton, Basil A. Smith, Latonia Taliaferro-Smith, Peri Nagappan, Clayton Yates, Majd Zayzafoon, Leland W. K. Chung, Valerie A. Odero-Marah

**Affiliations:** 1 Center for Cancer Research and Therapeutic Development and Department of Biological Sciences, Clark Atlanta University, Atlanta, Georgia, United States of America; 2 The Department of Hematology and Medical Oncology and Winship Cancer Institute, Emory University School of Medicine, Atlanta, Georgia, United States of America; 3 Department of Biology, Tuskegee University, Tuskegee, Alabama, United States of America; 4 The Department of Pathology, University of Alabama at Birmingham, Birmingham, Alabama, United States of America; 5 The Uro-Oncology Research Program, Department of Medicine, Samuel Oschin Comprehensive Cancer Institute, Cedars-Sinai Medical Center, Los Angeles, California, United States of America; Rajiv Gandhi Centre for Biotechnology, India

## Abstract

Snail transcription factor is up-regulated in several cancers and associated with increased tumor migration and invasion via induction of epithelial-to-mesenchymal transition (EMT). MAPK (ERK1/2) signaling regulates cellular processes including cell motility, adhesion, and invasion. We investigated the regulation of ERK1/2 by Snail in breast cancer cells. ERK1/2 activity (p-ERK) was higher in breast cancer patient tissue as compared to normal tissue. Snail and p-ERK were increased in several breast cancer cell lines as compared to normal mammary epithelial cells. Snail knockdown in MDA-MB-231 and T47-D breast cancer cells decreased or re-localized p-ERK from the nuclear compartment to the cytoplasm. Snail overexpression in MCF-7 breast cancer cells induced EMT, increased cell migration, decreased cell adhesion and also increased tumorigenicity. Snail induced nuclear translocation of p-ERK, and the activation of its subcellular downstream effector, Elk-1. Inhibiting MAPK activity with UO126 or knockdown of ERK2 isoform with siRNA in MCF-7 Snail cells reverted EMT induced by Snail as shown by decreased Snail and vimentin expression, decreased cell migration and increased cell adhesion. Overall, our data suggest that ERK2 isoform activation by Snail in aggressive breast cancer cells leads to EMT associated with increased cell migration and decreased cell adhesion. This regulation is enhanced by positive feedback regulation of Snail by ERK2. Therefore, therapeutic targeting of ERK2 isoform may be beneficial for breast cancer.

## Introduction

Breast cancer is the second most commonly diagnosed cancer, accounting for almost 1 in 3 cancers diagnosed in US women [1]. One of the main causes of mortality from cancer is metastasis [2]. Epithelial-Mesenchymal Transition (EMT) is a process that promotes tumor progression; Snail (snail1) transcription factor is a C2H2 zinc finger protein that promotes EMT, which is characterized by decreased expression of cell adhesion molecules such as E-cadherin, VE-cadherin, Claudins, Occludin, Desmoplakin, Cytokeratins, and Mucin-1, and increased expression of mesenchymal markers such as vimentin and N-cadherin [3], [4]. Snail can be induced by growth factors such as transforming growth factor beta (TGF-β) and epidermal growth factor (EGF) [3]. Snail has been shown to increase resistance to apoptosis in hepatocytes and Madine Darby Canine Kidney (MDCK) cells [3], [5]–[7]. Snail is induced by TGF-β and that upregulates pro-inflammatory interleukins and matrix metalloproteinases (MMPs), which help to degrade the extracellular matrix (ECM) [3]. This activity shifts the microenvironment to a more radical and invasive profile [6].

Modulation of Snail phosphorylation has been a hallmark of several studies on Snail-mediated EMT [8]–[10]. Active Snail is localized within the nucleus as a 264 amino acid transcriptional repressor of target promoters, and is phosphorylated by a p21-activated kinase 1 (PAK1) on Ser246 [11]. PAK1 phosphorylation of Snail activates Snail protein and promotes Snail accumulation within the nucleus to promote EMT. Snail is phosphorylated by glycogen synthase kinase-3β (GSK-3β) at two consensus motifs [12], [13]. GSK-3β phosphorylation induces β-TRCP-mediated ubiquitination, localization of Snail within the cytoplasm and subsequent proteasomal degradation of Snail [13]. Additionally, Snail phosphorylation by protein kinase D1 (PKD1) on Ser11 leads to nuclear export of Snail and EMT inhibition [14]. The zinc finger domain of Snail serves as its nuclear localization sequence and directs its movement into the nucleus [15]. Obtaining a better understanding of the signaling pathways involved in breast cancer will aid in developing more effective therapies and prevention strategies. Cross-communication between different pathways allows cells to identify and respond appropriately to the extracellular environment [16]. The receptor tyrosine kinases (RTK) like epidermal growth factor receptor (EGFR), Ras/Raf/MEK/ERK, and PI3K/AKT pathways are all involved in cancer development, progression, and metastasis. Snail has been strongly implicated in the Mitogen-activated protein kinase (MAPK) pathway in breast cancer cells [17].

MAPK and extracellular-regulated kinases 1 and 2 (ERK1/2) are significant signaling proteins that control several processes, including: proliferation, survival, motility, adhesion, invasion and survival [18]. The ERK1/2 cascade has several distinct functions that differ depending on its subcellular localization. Nuclear ERK1/2 activity (p-ERK) has been associated with malignant mammary tumors and poor prognosis [19], [20]. One study showed that chemokine (C-X-C) ligand 5 (CXCL5) could activate Raf/MEK/ERK, MSK1, Elk-1, and Snail, while E-cadherin was down-regulated in breast cancer cell lines [17].

MAPK pathway inhibition has proven to be a promising method of decreasing tumor growth by directly hindering cell survival [18]. However, the use of oral MEK inhibitor CI-1040 (800 mg) to treat non-small-cell lung, breast, colon, and pancreatic cancer has not been entirely successful [21]. Investigators reported a dramatic increase in PI3K/Akt activity after administration of CI-1040, indicating a shift in this pathway and potential tumor resistance. Further studies deem hopeful, especially with a second generation MEK inhibitor, PD 0325901 [21]. More importantly, MAPK pathway inhibition has focused on general MAPK inhibition without dissecting out the role of the different ERK isoforms.

We investigated the role of Snail in regulating MAPK in breast cancer cells and found that Snail and ERK1/2 activity was higher in breast cancer cells as compared to normal mammary epithelial cells. We have novel data that Snail overexpression in MCF-7 promotes nuclear ERK1/2 activation, resulting in Elk-1 activation and EMT; Snail could be further regulated by ERK2 isoform by a positive feedback mechanism. These findings begin to uncover nuclear ERK2 isoform activity as a specific subcellular target of therapy for aggressive breast cancer.

## Materials and Methods

### Cell Culture, Antibodies, and Reagents

The human breast cancer cells lines, T47-D, MCF-7, and MDA-MB-231 were obtained from ATCC, Manassas, VA. The MCF-7 cells stably transfected with empty Neo vector (MCF-7 Neo) and or constitutively active Snail (MCF-7 Snail) used for most of these studies were kindly provided by Dr. Mien-Chie Hung, The University of Texas MD Anderson Cancer Center, Houston TX, and established as described previously [14]. Cells were grown in RPMI medium supplemented with 10% fetal bovine serum and 1% penicillin/streptomycin, at 37°C with 5% CO_2_ in a humidified incubator. RPMI medium and penicillin/streptomycin (P/S) were purchased from VWR Int., West Chester, PA. Fetal bovine serum (FBS) and Charcoal/dextran treated FBS (DCC-FBS) were from Hyclone, South Logan, UT. Matched human tumor/normal breast cancer tissue lysates were purchased from (Protein biotechnologies, Ramona, CA). Anti-human β-actin antibody was from Sigma-Aldrich, Inc., St Louis, MO. Rat monoclonal anti-Snail antibody, HRP-conjugated goat anti-rat antibody, rabbit monoclonal anti-phospho-ERK antibody, rabbit polyclonal anti-ERK antibody, rabbit polyclonal anti-phospho-p90RSK (Thr359/Ser363), rabbit polyclonal anti-phospho-Elk-1 (Ser383), and rabbit monoclonal GAPDH (D16H11) antibodies were from Cell Signaling Technology, Inc., Danvers, MA. Mouse monoclonal E-cadherin, rabbit polyclonal p90RSK-1 (C-21), and rabbit polyclonal Elk-1 (I-20), mouse monoclonal vimentin (V9), and rabbit polyclonal topoisomerase I (H-300) antibodies were purchased from Santa Cruz Biotechnology, Inc. (Santa Cruz, CA). HRP-conjugated sheep anti-mouse, sheep anti-rabbit and the Enhanced chemiluminescence prime (ECL prime) detection reagent were purchased from Amersham Biosciences, Buckinghamshire, UK. Luminata Forte HRP chemiluminescence detection reagent was purchased from EMD Millipore (Billerica, MA). The protease inhibitor cocktail was from Roche Molecular Biochemicals, Indianapolis, IN. from BD Biosciences, San Jose, CA. Rat tail collagen type I and human fibronectin were from BD Biosciences (Bedford, MA). UO126 (MEK1/2 inhibitor) and MG132 were purchased from EMD Calbiochem (Billerica, MA).

### Ethics Statement for Use of Animals

This study was carried out in strict accordance with the recommendations in the Guide for the Care and Use of Laboratory Animals of the National Institutes of Health. All of the animal procedures were approved and performed in accordance with the Emory University Institutional IACUC guidelines.

### Animal Experiments

Four-week-old female athymic *nu/nu* mice (National Cancer Institute) were implanted subcutaneously with 17β-estradiol-sustained release pellets and subsequently injected subcutaneously with 2×10^6^ cells per mouse of Neo or Snail-overexpressing MCF-7 cells mixed 1∶1 volume with matrigel (BD Biosciences). There were 6 mice in each group. The mice were sacrificed after 2 weeks by carbon dioxide overdose followed by cervical dislocation, the tumors excised and tumor volume measured with a caliper (tumor volume was calculated as 3.14/6× largest diameter × smallest diameter squared). The tumors were used for histology studies and immunohistochemistry.

### Ethical Statement Related to the Use of Human Breast Tumor Samples

Breast tumors, and matched normal tissues were obtained from the following sources- a) Protein biotechnologies, Ramona, CA; b) US Biomax, Inc. (Catalog #BR1002, Rockville, MD). Protein Biotechnologies Inc. provides pharmaceutical, biotechnology, government, and academic institutions with human clinical specimen derivatives. Tissues are obtained through a global network of participating medical centers that employ IRB approved protocols and strict ethical guidelines to ensure patient confidentiality and safety. Identical procedures are used to prepare all patient samples. Specimens are flash frozen to −120°C within 5 min of removal to minimize autolysis, oxidation, and protein degradation. Tissue specimens are homogenized in modified RIPA buffer (PBS, pH 7.4, 1 mM EDTA, and protease inhibitors) to obtain the soluble proteins, and centrifuged to clarify.

### Tissue Microarray Analysis by Dual Immunofluorescence

The breast cancer and normal tissue microarray slide was deparaffinized with xylene, dehydrated with alcohol series from 100% ethanol to 50% ethanol, antigens were retrieved at 125°C for 30 seconds, peroxidases were blocked using 0.03% hydrogen peroxide, and blocked using either goat or rabbit sera. Primary antibodies (Snail anti-rat and p-ERK anti-rabbit) were added to the slide and incubated overnight at 4°C. After washing with 1× Tris Buffered Saline-Tween (TBS-T) and 1× phosphate buffered saline (PBS), secondary antibodies anti-rabbit Oregon green 488 (Invitrogen, Carlsbad, CA) and anti-goat Texas red (Vector Laboratories, Burlingame, CA) were added and incubated for 30 minutes in darkness. The slide was washed with 1× TBS-T, counterstained with DAPI to detect nuclei, washed briefly with double deionized water, and mounted using Fluorogel mounting medium (Electron Microscopy Sciences, Hatfield, PA). Fluorescence microscopy was performed using Zeiss (Axiovision Rel 4.8) and Apotome software.

### Immunohistochemistry

Paraffin-embedded tumor tissues were sliced into 5 µm thick sections and mounted on glass. The slides were deparaffinized and rehydrated through a graded series of ethanols to deionized water followed by antigen retrieval with Reveal Decloaker RTU antigen unmasking solution (Biocare Medical, Concord, CA). The slides were incubated with Snail primary antibody overnight at 4°C, washed and incubated with HRP-conjugated secondary antibody for 30 min at room temp. Slides were washed with TBS-T 3×5 min, 1× PBS 3×5 min, then incubated with an avidin+biotin solution from (Biocare Medical, Concord, CA). Slides were gently and briefly washed with 1× PBS two times; the first wash was for 5 min in a humidified chamber and the second wash was for 15 min with low agitation. Slides were incubated with 3,3-Diaminobenzidine (DAB) for no more than 2 min at room temp. Slides were washed with deionized water 3×5 min, dipped in hematoxylin/eosin for 3 min, rinsed in tap water for 5 min, and dehydrated in an up-graded series of ethanols. Slides were dipped in xylene and mounted using a xylene-based mounting media. Images of slides were taken using Zeiss microscope and Rel 4.8 software.

### Immunofluorescence Using Breast Cancer Cell Lines

5×10^3^ cells were plated into 16 well chamber slides (Bio-Tek, Nunc, Winooski, VT). For treatments, cells were either untreated, treated with control siRNA or Snail siRNA. Fixation was performed with methanol/ethanol 1∶1 volume for 5 min, followed by washes with 1× PBS and blocking with protein blocking solution without serum (Dako, Camarillo, CA) for 10 min at room temp. Subsequently, slides were incubated with primary antibody at 1∶50 or 1∶100 dilutions in Dako antibody diluent solution for 1 h at room temp. Slides were washed with 1× TBS-T (Dako, Camarillo, CA), then incubated with secondary antibody in the dark for 1 h at room temp. Secondary antibodies used were anti-rabbit Oregon green 488, anti-mouse Alexa red 594 (Invitrogen, Carlsbad, CA) or anti-goat Texas red (Vector Laboratories, Burlingame, CA). Slides were washed with 1× TBS-T and double deionized water, prior to counterstaining with DAPI (1 µg/ml, Santa Cruz Biotechnology, Santa Cruz, CA). Slides were mounted using Fluorogel mounting medium (Electron Microscopy Sciences, Hatfield, PA). Fluorescence microscopy was performed using Zeiss microscope and Axiovision Rel 4.8 software.

### Short Interfering RNA Transfection (siRNA)

Transient transfections were performed with 100 nM of non-silencing ON-TARGET (Catalog #D-001810-10) or ON-TARGETplus siRNA (Thermo Scientific - Dharmacon, Lafayette, CO) for Snail, ERK 1 isoform and ERK 2 isoform, as per the manufacturer's instructions. Briefly, T47-D, MDA-MB-231, MCF-7 Neo, and MCF-7 Snail cells were seeded overnight in 6-well dishes then incubated with either non-silencing or siRNA against Snail, ERK1 or ERK2 in phenol-free RPMI without FBS or antibiotics for 5 hours; subsequently the media were replaced with 5% DCC phenol-free RPMI for an additional 72 hours. Lysates from whole cell, nuclear and cytoplasmic extracts were harvested and quantitated for respective experiments.

### Stable Snail Transfection in MCF-7

In order to confirm our findings from MCF-7 Neo and MCF-7 Snail cells obtained from Dr Mien-Chie Hung, we generated our own stable cell lines as follows: Parental MCF-7 cells grown to 90% confluence in a 6 well culture dish were transfected with either 1 µg of Snail cDNA or the empty neomycin vector (Neo), using Lipofectamine 2000 according to manufacturer instructions. (Invitrogen, San Diego, CA). Stable transfectants were selected by treatment with 800 µg/ml of G418 and maintained in 400 µg/ml G418. Selected clones were tested for Snail expression by Western blot analysis.

### Western Blot Analysis

Cells were lysed in a modified RIPA buffer (50 mM Tris pH 8.0, 150 mM NaCl, 0.02% NaN_3_, 0.1% SDS, 1% NP-40, 0.5% sodium deoxycholate) containing 1.5× protease inhibitor cocktail, 1 mM phenylmethylsufonyl fluoride (PMSF), and 1 mM sodium orthovanadate. Whole cell lysates were freeze-thawed at −80°C/4°C for three cycles, then centrifuged at 13,500 rpm for 30 min at 4°C. Supernatants were collected and quantified using a micro BCA assay (Promega, Madison, WI). 30–50 µg of cell lysates were resolved using 10% SDS PAGE, followed by transblotting onto nitrocellulose membrane (Bio-Rad Laboratories, Hercules, CA). Membranes were blocked in 5% milk (TBS with 0.05% Tween-20, 0.05% BSA containing 5% milk) or 3% milk (TBS-T containing 3% milk), then washed and incubated with primary antibody dilution buffer. After washing, the membranes were incubated in peroxidase-conjugated sheep anti-mouse, sheep anti-rabbit, or goat anti-rat IgG, washed, and visualized using ECL prime reagent (GE Healthcare, Buckinghamshire, UK) or Luminata Forte ECL reagent (Millipore, Billerica, MA). The membranes were stripped using Restore Western blot stripping buffer (Pierce Biotechnology, Inc., Rockford, IL) prior to re-probing with a different antibody.

### *In vitro* Cell Migration Assay

We utilized Costar 24-well plates containing a polycarbonate filter insert with an 8- µm pore size, to coat with 3.67 µg/ µl rat tail collagen I or 2.5 µg/cm^2^ human fibronectin on the outside for 24 h at 4°C. 5×10^4^ cells were plated in the upper chamber containing RPMI supplemented with 0.1% fetal bovine serum (FBS), whereas the lower chamber contained RPMI supplemented with 10% FBS. After 5 h, cells that migrated to the bottom of the insert were fixed, stained with 0.05% crystal violet, and counted to obtain the relative migration.

### *In vitro* Cell Adhesion Assay

96 well plates were coated with 3.67 µg/ µl of rat-tail collagen I or 2.5 µg/cm^2^ of fibronectin overnight at 4°C. Binding sites were blocked with 0.1% bovine serum albumin (BSA) in PBS followed by plating of 3×10^4^ cells in complete RPMI. After incubation for 20 min, cells were treated with Percoll flotation medium and Percoll fixative for 15 min at room temp, washed with 1× PBS and stained with 0.05% crystal violet. The following day, each well was solubilized with Sorenson solution, and OD read at 590 nm using a Gen5 automated plate reader to quantify relative cell adhesion.

### Subcellular Fractionation

Subcellular fractionations were performed per the manufacturer's instructions (Thermo Scientific). Briefly, cells at 80–90% confluence were lysed in a series of buffers containing protease inhibitors (25X) with CERI (100 µl), CERII (5.5 µl), or NER (50 µl). Centrifugation steps were performed to obtain a non-nuclear fraction and an intact nuclear pellet, followed by further lysing to isolate the nuclear fraction. 50 µg of non-nuclear and nuclear fractions were utilized for Western blot analysis. Mouse anti-topoisomerase I and mouse anti-GAPDH antibodies were used to ensure the integrity of nuclear and non-nuclear fractions, respectively.

### Statistical Analysis

Data were analyzed by a paired student's t-test or ANOVA using GraphPad Prism software. ImageJ software was used to quantitate densitometry. P values less than 0.05 were considered statistically significant.

## Results

### p-ERK Is Increased in Patient Breast Tumor Tissues

**Figure 1:**
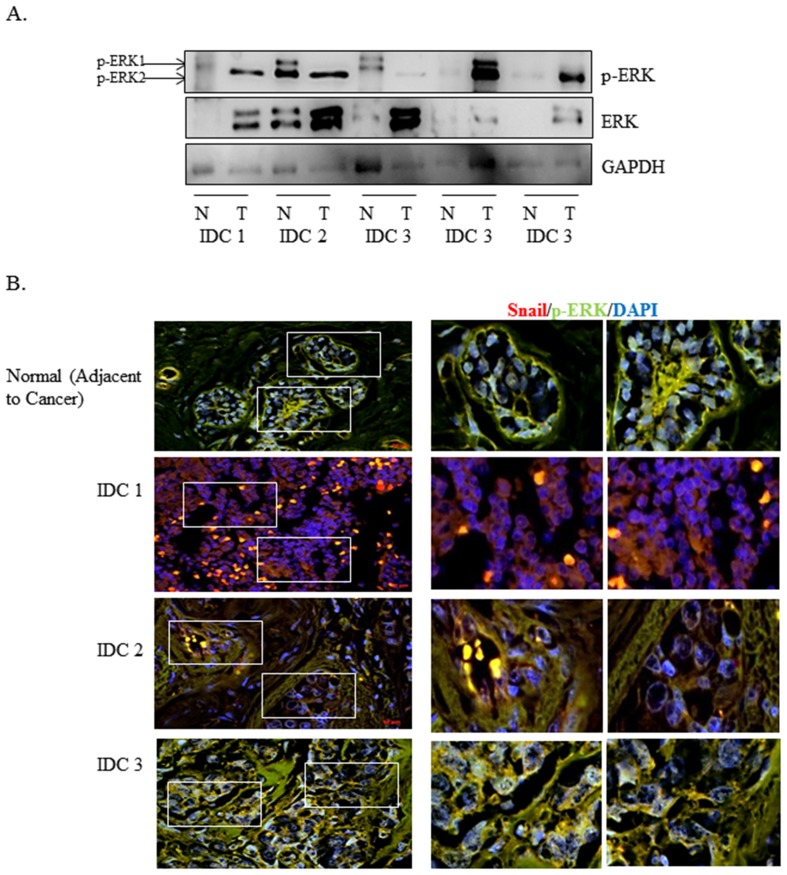
p-ERK expression is increased in patient tumor tissues. (A) 10 µg of normal/tumor-matched infiltrating ductal carcinoma (IDC) grades 1–3 and lymph node metastatic patient lysates were separated using SDS-PAGE electrophoresis, then immunoblotted onto nitrocellulose. Expression of p-ERK and ERK was determined using Western blot analysis. β-actin was used as Western blot loading control. (B) Human breast cancer tissue microarray was double-labeled with p-ERK (green) and Snail (red) antibodies using immunofluorescence analysis. DAPI was used to identify the nuclei. Images were captured using Zeiss Axiovision Rel4.8 at 20× (left panel) and Apotome software at and 40× oil magnification (right panel). Results are representative of at least three independent experiments.

We analyzed the expression of p-ERK and total ERK in normal/tumor matched patient lysates using Western blot analysis. Most of the tumor lysates expressed higher levels of p-ERK as compared to normal tissue (Figure **1A**). We analyzed the expression of p-ERK and Snail using a human tissue microarray containing normal and tumor tissue cores from patients. We found that p-ERK was predominantly cytoplasmic in normal epithelial tissue, while Snail was low (Figure **1B**, Figure **S1**). The higher grade tumor samples expressed a higher level of Snail and more nuclear expression of p-ERK within epithelial cells, which co-localized with Snail, as well as high p-ERK within the stroma (Figure **1B**, Figure **S1**). This confirms previous studies that p-ERK is increased in breast cancer, while suggesting that Snail may co-localize with p-ERK in breast cancer.

### Snail Expression Is Associated with Increased p-ERK in Breast Cancer Cells

**Figure 2:**
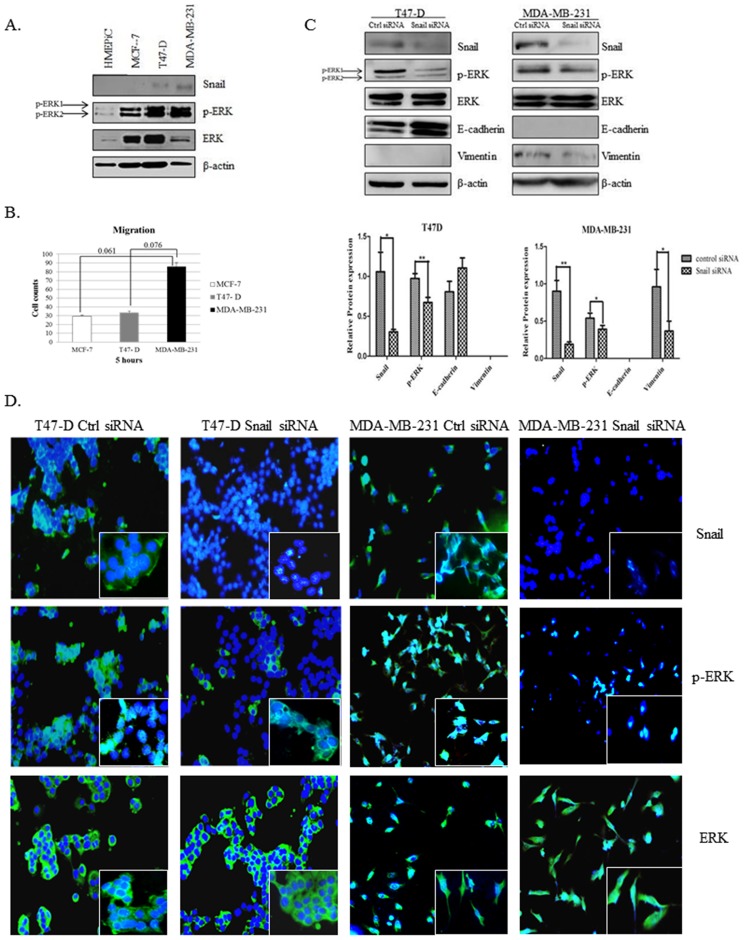
Snail is correlated with increased p-ERK in breast cancer cells. (A) Normal immortalized breast epithelial cells (HMEPiC), the adenocarcinoma cell lines MCF-7 and T47-D, and triple negative cell line MDA-MB-231 were utilized to analyze Snail, p-ERK and total ERK protein levels by Western blot analysis. (B) MCF-7, T47-D, and MDA-MB-231 cell lines were utilized to perform a migration assay on collagen. Cells that had migrated through the boyden chamber insert were fixed, stained, counted and graphed. Bars, SD, paired Student's t-test. (C) T47-D and MDA-MB-231 cells were transiently transfected with control siRNA or Snail siRNA and expression levels of Snail, p-ERK, and ERK were determined with Western blot analysis (top panel). Relative protein levels compared to actin were analyzed by Image J analysis using National Institute of Health (NIH) software and plotted (bottom panel). Bars, SD. Statistical analysis was done using ANOVA and Tukey's Multiple Comparison as Post Hoc (* *p*≤0.05, ** *p*≤0.01). (D) Immunofluorescent analysis was also performed on T47-D and MDA-MB-231 cells transiently transfected with control siRNA or Snail siRNA. β-actin was utilized as a loading control for Western blot analysis; DAPI was used to identify the nuclei in immunofluorescent analyses. Magnification 20X; Inset 40X. Results represent experiments performed in triplicate at least twice independently.

We examined the expression of Snail transcription factor in a panel of normal breast epithelial and breast cancer cells lines of increasing aggressiveness by Western blot analysis. The normal breast epithelial cells (HMEPiC) and MCF-7 breast cancer cell line did not express detectable levels of Snail, while Snail was detectable in breast cancer cell lines T47-D and higher in MDA-MB-231, which displayed the highest cell migratory potential (Figure **2A, B**). p-ERK was also increased in breast cancer cell lines as compared to normal breast epithelial cells (Figure **2A**). This demonstrates that Snail expression positively associates with p-ERK and cell migration. To determine the effects of Snail knockdown on p-ERK in T47-D and MDA-MB-231 breast cancer cells, we transiently transfected these cells with control siRNA or Snail siRNA and analyzed the expression of Snail, p-ERK, and EMT markers (E-cadherin and vimentin) by Western blot and immunofluorescence analyses. Snail knockdown decreased p-ERK significantly in MDA-MB-231 and T47-D cells (Figure **2C, D** and Figure **S2**). Interestingly, there was also a marked re-localization of p-ERK from the nucleus to the cytoplasm upon Snail knockdown in T47-D cells, as seen by immunofluorescence (Figure **2D**, Figure **S2**). Additionally, Snail knockdown increased levels of E-cadherin in T47-D and decreased levels of vimentin in MDA-MB-231 cells, suggesting a partial reversion of EMT (Figure **2C**). These data demonstrate that Snail and p-ERK increase with breast cancer progression and that Snail may regulate ERK activity in breast cancer cells.

### Snail Increases EMT *In Vitro* and Tumorigenicity *In Vivo*

**Figure 3:**
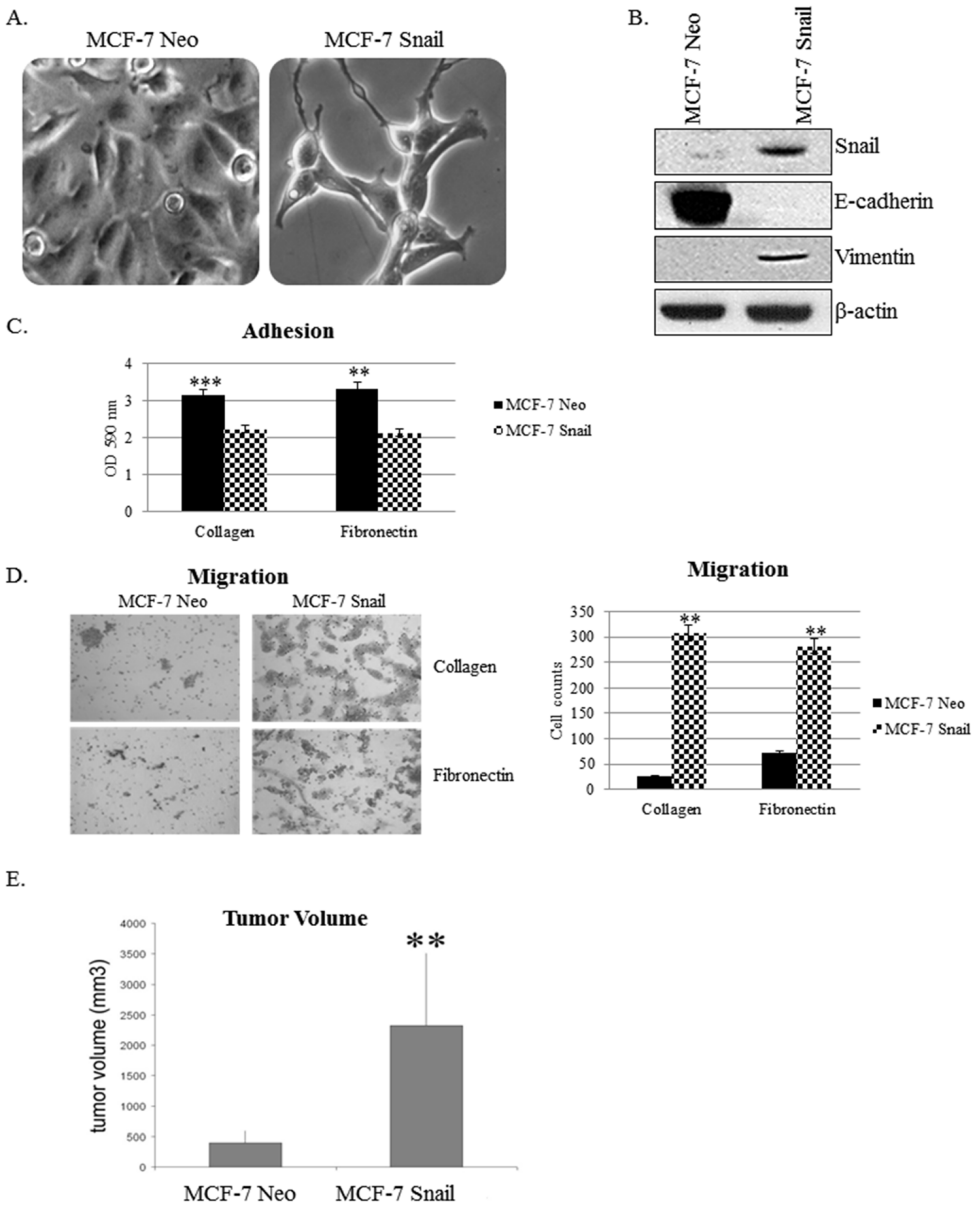
Snail increases EMT *in vitro* and tumorigenicity *in vivo*. (A) Morphology images of MCF-7 Neo and MCF-7 Snail cells were captured by brightfield microscopy (100× magnification). (B) Expression of EMT markers Snail, E-cadherin, and Vimentin was analyzed using Western blot. (C) Adhesion and (D) migration assays performed on collagen I and fibronectin matrices were imaged at 10× magnification (left panel), counted and graphed (right panel). (E) MCF-7 Neo or MCF-7 Snail cells were injected subcutaneously into nude female mice (N = 6) and two weeks later, mice were sacrificed and tumor volumes measured and graphed. β-actin was utilized as a loading control for Western blot analysis. Statistical Analysis was done using paired Student's t-test, Bars, SD (**p<0.01, ***p<0.001). Results are representative of at least three independent experiments.

Since hormone-dependent MCF-7 cells expressed barely detectable levels of Snail, we utilized MCF-7 cells that have been transfected with either the Neo empty vector or constitutively active Snail cDNA for *in vitro* and *in vivo* studies. The MCF-7 Neo/MCF-7 Snail cell model has been used as an EMT progression model for breast cancer [14]. We confirmed that MCF-7 Neo cells maintain a more epithelial and cuboidal morphology, which was completely transformed to a more mesenchymal and fibroblast-like morphology in MCF-7 Snail cells (Figure **3A**). We also confirmed by Western blot analysis that this model represents an EMT model by analyzing the expression of EMT markers; MCF-7 Snail cells demonstrated higher levels of Snail and vimentin and lower levels of E-cadherin as compared to MCF-7 Neo cells (Figure **3B**). We also analyzed the effects of Snail overexpression on cell adhesion and migration using rat tail collagen I and human fibronectin matrices, and found that MCF-7 Snail cells displayed decreased cell adhesion and increased cell migration on both matrices (Figure **3C, 3D**). To determine the effects of Snail overexpression *in vivo*, we injected MCF-7 Neo and MCF-7 Snail cells subcutaneously into female nude mice. Significantly larger tumor volumes were observed in MCF-7 Snail tumor xenografts as compared to MCF-7 Neo xenografts after 2 weeks (Figure **3E**). H&E staining was performed as well as immunohistochemistry to demonstrate higher expression of Snail in MCF-7 Snail tumor xenograft tissues as compared to MCF-7 Neo tumor xenografts (Figure **S3**). Therefore, Snail increases EMT and tumorigenicity in MCF-7 breast cancer cells.

### Snail Promotes Nuclear Translocation of p-ERK

**Figure 4:**
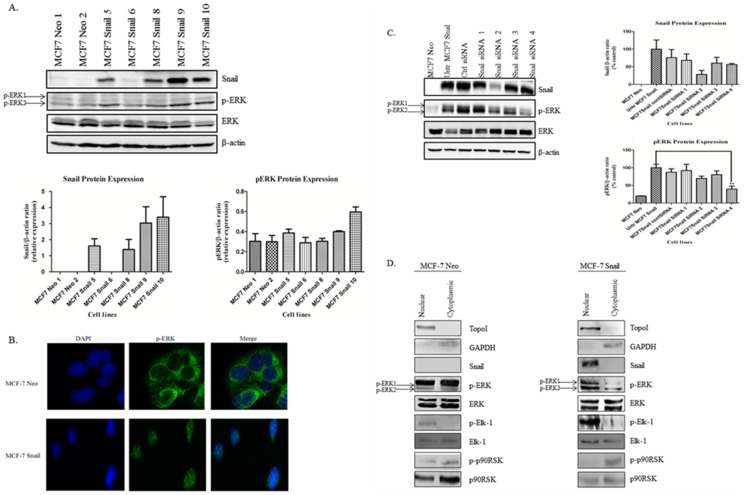
Snail promotes nuclear ERK activation. (A) MCF-7 parental cells were stably transfected with constitutively active Snail cDNA utilizing lipofectamine 2000, and stable clones selected using G418 and isolated. Expression of Snail, p-ERK and ERK in representative Neo and Snail clones was analyzed by Western blot analysis (top panel) and quantified (bottom panel). (B) Expression and localization of p-ERK in MCF-7 Neo and MCF-7 Snail cells was analyzed using immunofluorescence. (C) MCF-7 Snail cells were transiently transfected with either control or Snail siRNA for 72 h and cell lysates analyzed for expression of Snail, p-ERK and ERK by Western blot analysis (left panel) and results of Western blots were quantified and graphed (right panel). (D) Nuclear and cytoplasmic extracts were isolated from MCF-7 Neo and MCF-7 Snail cells. The expression of Snail, p-ERK, ERK, p-Elk-1, Elk-1, p-p90RSK, and p90RSK was analyzed by Western blot analysis. β-actin was utilized as a loading control for Western blot analysis; DAPI was used in immunofluorescence to identify the nuclei. Magnification, 63× oil. Topoisomerase I (Topo I) was utilized as a nuclear marker while GAPDH was utilized as a cytoplasmic marker. Relative protein levels compared to actin was analyzed by Image J analysis using National Institute of Health (NIH) software and plotted. Values were expressed as mean ± S.E.M. Statistical analysis was done using ANOVA and Tukey's Multiple Comparison as Post Hoc (** *p*≤0.01). Results are representative of experiments performed in triplicate.

Next, we examined p-ERK levels upon Snail overexpression. For this, we retransfected parental MCF-7 cells stably with constitutively active Snail cDNA or empty Neo vector and selected various clones of Neo and Snail. Using representative clones, we observed that MCF-7 Snail clones that expressed high levels of Snail also expressed higher p-ERK as compared to MCF-7 Neo clones (Figure **4A**). Interestingly, Snail overexpression correlated with predominantly nuclear localization of p-ERK as observed by immunofluorescence, while p-ERK was predominantly cytoplasmic, surrounding the nucleus in MCF-7 Neo cells (Figure **4B**, Figure S**4**). We further confirmed that Snail could regulate p-ERK by knocking down Snail in a representative MCF7-Snail clone; this led to decreased p-ERK (Figure **4C**). We further examined p-ERK localization utilizing Western blot analysis of nuclear and cytoplasmic fractions. We observed that Snail overexpression led to p-ERK primarily within the nuclear fraction as compared to the cytoplasmic fraction (Figure **4D**). However, in MCF-7 Neo cells, although there was p-ERK in both the nuclear and cytoplasmic fractions, it was interesting to note that it was predominantly the p-ERK1 isoform (Figure **4D**). Furthermore, we noted that in MCF-7 parental cells, p-ERK staining was similar to MCF-7 Neo cells and co-localized with nuclear import protein nucleoporin98 (NUP98), a nuclear membrane marker, suggesting that the predominant nuclear expression of p-ERK within these cells may reside within the nuclear membrane (data not shown). To determine whether Snail-mediated nuclear translocation of p-ERK may be associated with downstream effectors of p-ERK, we reviewed literature about MAPK and its effectors within the subcellular compartments and found that p90 ribosomal s6 kinase (p90RSK) is a cytoplasmic substrate of p-ERK, while Elk-1 transcription factor is a nuclear substrate of p-ERK [23]. P90RSK and Elk-1 are involved in tumor progression and biochemical changes in chromatin structure, and indirectly increasing cell proliferation through c-Fos activation, respectively [23]. We analyzed the protein expression of phosphorylated (active) and total forms of p90RSK and Elk-1 in MCF-7 Neo and MCF-7 Snail cells. We found that the p-p90RSK was expressed predominantly within the cytoplasmic fractions of both MCF-7 Neo and MCF-7 Snail cells; however, p-Elk-1 was highly expressed predominantly in the nuclear compartment and at significantly higher levels in MCF-7 Snail as compared to MCF-7 Neo cells (Figure **4D**). Further evidence that Snail may promote activation of ERK1/2, which subsequently activates Elk-1 was supported by the fact that Snail knockdown in MDA-MB-231 cells led to translocation of p-Elk-1 from the nucleus into the cytosol (Figure **S5**).

### Inhibition of ERK Activity with UO126 Decreases Snail Expression and Partially Reverts EMT Independent of Proteasomal Degradation

**Figure 5:**
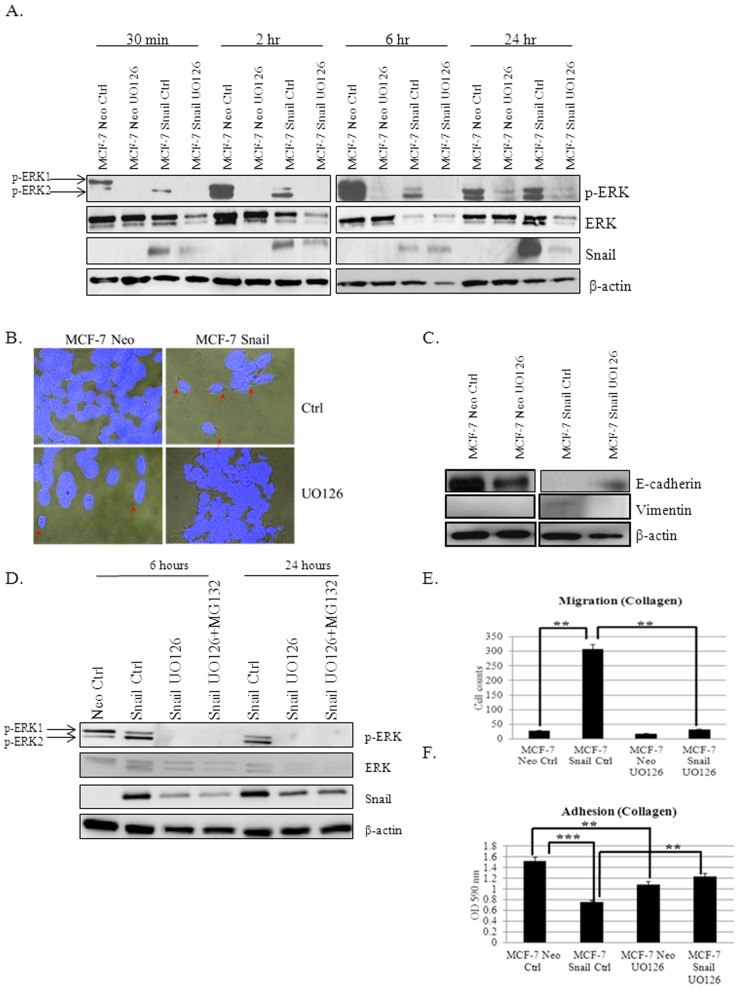
Inhibition of p-ERK with UO126 decreases Snail and reverts EMT independent of proteasomal degradation. (A) Expression of p-ERK, ERK and Snail was analyzed by Western blot analysis in MCF-7 Neo and MCF-7 Snail cells treated with DMSO control (Ctrl) or UO126 for 30 min, 2 h, 6 h, and 24 h. (B) MCF-7 Neo and MC-7 Snail cells were treated with either DMSO (control) or UO126 and stained with DAPI. Cell morphology and integrity were analyzed by merging DAPI immunofluorescence imaging with brightfield microscopy. (C) Expression of E-cadherin and vimentin in cells treated with DMSO Ctrl or UO126 was analyzed by Western blot analysis. (D) MCF-7 Snail were treated for 6 h and 24 h with DMSO Ctrl, UO126 and UO126+MG132. Cell lysates were analyzed by Western blot analysis. (E) Migration and (F) adhesion assays was performed on MCF-7 Neo and MCF-7 Snail cells treated with DMSO Ctrl or UO126. β-actin was utilized as a loading control for Western blot analysis; DAPI was used to identify the nuclei in immunofluorescence analyses. Maginification 40X. Statistical Analysis was done using ANOVA and Tukey's Multiple Comparison as Post Hoc (**p≤0.01, ***p≤0.001). Values were expressed as mean ± S.E.M (N = 3). Results are representative of at least three independent experiments.

MAPK/ERK signaling is closely involved with cancer development, progression and metastasis [18], [23], [27]. We sought to determine if Snail-mediated EMT may be regulated via p-ERK in our model of breast cancer. To accomplish this task, we inhibited p-ERK using MEK inhibitor UO126 (20 µM) for 30 min, 2 h, 6 h, and 24 h. MCF-7 Neo and MCF-7 Snail cells treated with UO126 led to p-ERK inhibition within 30 min up to 24 h (Figure **5A**). Interestingly, Snail expression was decreased in MCF-7 Snail cells within 24 h after UO126 treatment (Figure **5A**). Also noted, was the dramatic changes in MCF-7 Snail cell morphology after UO126 treatment; the cells became more epithelial and clumped after 24 h and were still viable as shown by DAPI staining ( Figure **5B** ). EMT was antagonized as shown by decreased vimentin and increased E-cadherin expression ( Figure **5C** ), which was associated with significant decrease in cell migration and increase in cell adhesion (Figure **5E**). To determine whether ERK inhibition was inducing proteasomal-mediated degradation of Snail, we pre-treated MCF-7 Neo and MCF-7 Snail cells with MG132 proteosomal degradation inhibitor for 2 h prior to treatment with UO126 for 6 or 24 h. We observed that inhibition of Snail expression following UO126 treatment was not restored by MG132 (Figure **5D**), suggesting that UO126 does not decrease Snail protein expression *via* the proteosomal pathway. Conversely, we observed that UO126 treatment of MCF-7 Neo cells altered morphology and led to spindle–shaped cells with extensions (Figure **5B**, arrows), which was accompanied by decreased expression of E-cadherin (Figure **5C**), as well as decreased cell adhesion (Figure **5E**). These data suggest that although Snail can regulate p-ERK, there may also be a feedback loop by which p-ERK can also positively regulate Snail expression. Furthermore, it appears that p-ERK inhibition in MCF-7 Snail cells reverts EMT, while surprisingly, p-ERK inhibition in MCF-7 Neo cells antagonizes cell adhesion.

### ERK2 Isoform Regulates Snail Expression and EMT

**Figure 6:**
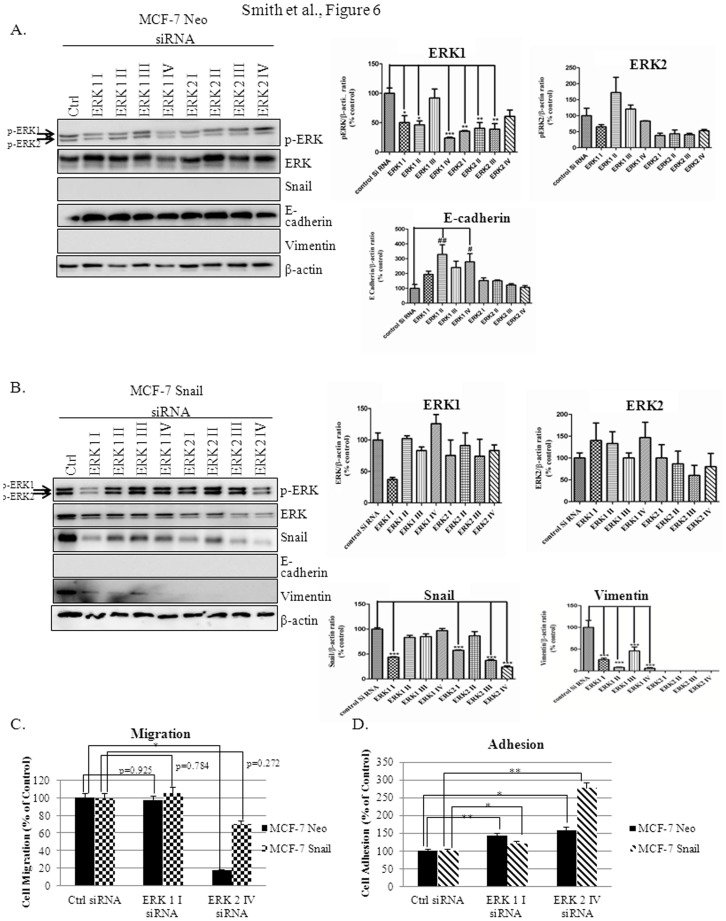
ERK2 isoform regulates Snail expression and EMT. (A) MCF-7 Neo and (B) MCF-7 Snail cells were transiently transfected with various constructs (I, II, III, V) of ERK1 and ERK2 siRNA for 72 h. Expression of p-ERK, ERK, Snail, E-cadherin, and vimentin was analyzed using Western blot (left panel). Relative protein levels compared to actin were analyzed by Image J analysis using National Institute of Health (NIH) software and plotted with Control siRNA set at 100% (right panel). (C) Migration and (D) adhesion assays were performed utilizing MCF-7 Neo and MCF-7 Snail cells transiently transfected with ERK1 I or ERK2 IV siRNA. β-actin was utilized as a loading control for Western blot analysis. Statistical Analysis was done using ANOVA and Tukey's Multiple Comparison as Post Hoc (*p≤0.05, **p≤0.01, ***p≤0.001; ^#^p≤0.05, ^##^p≤0.01). Values were normalized to untreated controls and expressed as mean ± S.E.M (N = 3). Results are representative of at least three independent experiments.

Since we had observed that MCF-7 Neo cells expressed a higher level of p-ERK1 isoform in both the cytoplasm and nuclear fraction, whereas MCF-7 Snail cells expressed a higher level of p-ERK1 and ERK2 isoform within the nuclear fraction, we dissected the roles of the different isoforms further. In MCF-7 Neo cells, we observed that neither ERK1 nor ERK2 siRNA affected Snail or vimentin expression, though E-cadherin levels appeared to increase with ERK1 knockdown (Figure **6A**). However, in MCF-7 Snail cells, both ERK1 and ERK2 siRNA decreased Snail and vimentin levels, but ERK2 siRNA decreased Snail and vimentin more significantly; however, E-cadherin was not affected (Figure **6B**). Functionally, ERK2 siRNA decreased cell migration in both MCF-7 Neo and MCF-7 Snail cells more significantly as compared to ERK1 siRNA (Figure **6C**). Moreover, ERK2 siRNA was also more effective than ERK1 siRNA at increasing cell adhesion in MCF-7 Snail cells (Figure **6D**). This suggests that ERK2 may be the primary regulator of Snail-mediated EMT when compared to ERK1.

## Discussion

Our research focused on the physiological functions influenced by Snail transcription factor to promote to breast cancer progression. Snail regulates epithelial-mesenchymal transition (EMT), which involves a loss of epithelial markers like E-cadherin and an increase in mesenchymal markers like vimentin [2], [3], [5]. Snail transcriptionally represses genes by binding to the enhancer sequence (E-box) of genes such as E-cadherin, Occludin, Claudins, and Mucin-1 [2], [3]. Moreover, Snail induces resistance to cell death, noted in skin tumors induced in mice, biopsies of breast carcinomas from patients, gastric cancer, and hepatocellular carcinomas [6]. This communication studied the relationship between Snail and phosphorylated ERK (p-ERK), in an effort to discover novel pathways by which ERK1/2 may be altered during breast cancer progression.

ERK1/2 is involved in several functions of breast cancer progression; ERK1/2 can regulate cell proliferation, survival, motility, and differentiation by indirectly shifting normal mammary epithelial cells into a more mesenchymal, less adherent state [22]. Previous studies have indicated an evident link between Snail, other Snail gene members (i.e., Snail, Slug, Twist) and MAPK/ERK signaling [2], [18], [23]. Snail has been shown to up-regulate p-ERK which subsequently increases cell survival in Madine Darby Canine Kidney (MDCK) cells [6]. Maintenance of Slug (Snail2) gene expression promotes tumor motility through the ERK signaling pathway in breast cancer [24]. Snail overexpression in ARCaP prostate cancer cells increased ERK activity, which was associated with increased cell migration and decreased cell adhesion [23].The present study associates Snail expression and p-ERK with breast cancer progression, as Snail protein and ERK activity was increased in breast cancer patient tissue as well as breast cancer cell lines (MCF-7, T47-D) and triple negative breast cancer cells (MDA-MB-231). MDA-MB-231 breast cancer cells are highly aggressive and migratory, as previously published [25] as well as in our study.

The expression of p-ERK and Snail were higher and co-localized in higher grade tumors. These findings correspond to a recent communication that analyzed p-ERK expression in patient tumors [26]. High levels of p-ERK were associated with poor prognosis based on poor differentiation, larger tumor sizes, and higher stages of breast cancer. Transient Snail knockdown using siRNA decreased p-ERK levels markedly in MDA-MB-231 cells, and led to re-localization of p-ERK from the nucleus to the cytoplasm in T47-D cells. Interestingly, the decreased p-ERK levels in MDA-MB-231 cells following Snail knockdown was accompanied by a shift in localization of p-Elk-1, a downstream substrate of nuclear p-ERK, from the nucleus to the cytoplasm. This would suggest that Snail may regulate p-ERK, and more specifically regulate its localization in cancer cells. Subcellular localization of ERK1/2 and its ability to undergo nucleocytoplasmic translocalization within the cell has been the focus of several investigations [27]. Although ERK1/2 is found abundantly throughout the cell in many organelles and cell structures, entry of this protein into the nucleus is highly selective; one study showed that nuclear entry of MAPK does not occur in primary ovarian and mammary epithelial cells due to lower import activity for ERK1/2 as compared to cancer cells [28]. These findings suggest that targeting nuclear MAPK may be an appropriate method to diagnose and/or treat cancer.

High levels of Snail expression and nuclear expression of ERK1/2 have been shown in separate studies to correlate with poor prognosis and cancer cell growth [2], [29]. We utilized the MCF-7 Neo/MCF-7 Snail cell model to more closely study the relationship between Snail expression and p-ERK. We confirmed that MCF-7 Neo/MCF-7 represented an EMT model; indeed, we found that when Snail is overexpressed in less invasive MCF-7 cells, the morphology of these cells shifted from epithelial to mesenchymal and fibroblastic. Snail overexpression also decreased E-cadherin epithelial marker while vimentin mesenchymal marker was increased, which was concomitant with decreased cell adhesion and increased cell migration. Additionally, Snail overexpression increased tumorigenicity *in vivo*. This study reports evidence for the first time, that Snail oncogene can negatively regulate adhesion to fibronectin and collagen in breast cancer cells, while also regulating other properties of epithelial cells that have been previously studied (i.e., E-cadherin reduction and increased migration). Our previous studies have shown that similarly in prostate cancer cells, Snail can decrease cell adhesion and increase cell migration by regulating integrin signaling, and down-regulating maspin tumor suppressor [23], [30].

The ERK/MSK1/Elk-1/Snail signaling pathway has been associated with breast cancer progression *in vitro* and *in vivo*; exogenous chemokine (C-X-C motif) ligand 5 (CXCL5) mimicked the effect of breast tumor-associated osteoblast (TAOBs) activities to induce Raf/MEK/ERK mediated up-regulation of Snail in MCF-7 and MDA-MB-231, which resulted in decreased E-cadherin expression [18]. We utilized our MCF-7 Neo/MCF-7 Snail cell model to more closely dissect out MAPK regulation by Snail. We found that Snail overexpression in MCF-7 breast cancer cells led to increased p-ERK levels, whose localization was predominantly within the nucleus. Moreover, the MCF-7 Snail cells with nuclear p-ERK also displayed increased activity of its downstream substrate, Elk-1. A previous study examined expression of p-ERK in a total of 886 breast cancer patients and found that surprisingly, p-ERK correlated inversely with tumor size [31]. However, they did not dissect out the localization of these low levels of p-ERK. It is possible that some tumors with low levels of p-ERK may actually have it localized within the nucleus, which may allow sufficient ERK activity that correlates with poor prognosis. These findings suggest that there may be a shift in MAPK localization as breast cancer cells become more metastatic. Surprisingly, MCF-7 Neo cells did express some p-ERK, but it was noted that it was mostly due to the ERK1 isoform, which was localized in the cytoplasm and the nucleus. However, we observed that the activity of nuclear Elk-1 was minimal in MCF-7 Neo cells, suggesting that p-ERK within these cells do not signal in the nucleus via Elk-1. Previously, it has been shown that up-regulation of ERK2 isoform activity contributed to increased invasion in MDA-MB-231 cells, which was also corroborated by 3D cell migration assays [32]. It is plausible that Snail may be able to activate preferentially the ERK2 isoform, which may lead to Elk-1 activation. However, we cannot totally rule out Snail activation of ERK1 isoform, which was also higher in nuclear extracts as compared to cytoplasmic extracts.

Interestingly, though we showed that Snail can regulate p-ERK, MAPK inhibition utilizing UO126 in MCF-7 Snail cells led to decreased expression of Snail, reversion of EMT as evidenced by increased E-cadherin, decreased vimentin, decreased cell migration and increased cell adhesion. Normally, the molecular half-life of Snail is only about 25 min, and is highly unstable [14]. The molecular mechanism that drives tightly-controlled Snail activation can include GSK-3β-directed phosphorylation that can include β-TrCP-directed ubiquitination and eventually proteasomal degradation [12], [14]. We investigated whether proteasomal degradation was the basis behind decreased Snail protein upon inhibition of MAPK activity, by pre-treatment with MG132 proteosomal degradation inhibitor. However, we found that MG132 did not prevent the loss of Snail protein upon UO126 treatment, suggesting that MAPK inhibition does not target Snail for proteosomal degradation. It has been reported that AP-1 activation can be induced by cellular stress brought on by UV-irradiation [33]. This mode of AP-1 activation induces Snail expression through the MAPK pathway in keratinocytes. Therefore, we propose that AP-1 activation may be an alternative pathway by which MAPK may regulate Snail and subsequently EMT. Moreover, we propose that Snail may promote EMT in breast cancer cells possibly through activation of p-ERK and preferentially the ERK2 isoform and not the ERK1 isoform since MCF-7 Neo cells have high levels of p-ERK1 that does not activate Elk-1 activity. In fact, inhibition of MAPK activity in MCF-7 Neo cells led to decreased E-cadherin expression and decreased cell adhesion; this is opposite to the effects observed in MCF-7 Snail cells.

We studied a possible mechanism of differential activation of the ERK1 and ERK2 isoforms, and their downstream targets during EMT. ERK1 or ERK2 siRNA were used to analyze the effects on EMT in MCF-7 Neo and MCF-7 Snail cells. ERK2 siRNA more markedly decreased Snail and vimentin expression in MCF-7 Snail cells, which correlated with decreased migration and increased adhesion when compared to ERK1 siRNA; however, E-cadherin expression was not restored. This differs from the effect of UO126, which was able not only to decrease Snail and vimentin expression, but to also restore E-cadherin expression. Additionally, while UO126 in MCF-7 Neo cells seemed to decrease E-cadherin and increase cell adhesion, neither ERK1 nor ERK2 siRNA was able to significantly affect cell migration or cell adhesion in MCF-7 Neo cells. These differences may be explained either by the level of knockdown (it was not complete with the siRNA but there was complete inhibition of p-ERK with UO126) or possibly due to the effect of UO126 on other proteins apart from ERK1/2. Therefore, our data suggest that although Snail can regulate p-ERK, there is also a feedback loop by which p-ERK, particularly the ERK2 isoform can positively regulate Snail expression. This would corroborate previous studies showing that increased ERK2 isoform activity contributed to increased invasion and migration in MDA-MB-231 [32].

Although most studies usually study ERK1/2 activity without dissecting out the role of each isoform, recent studies are suggesting that they may have different functions. For example, it has recently been shown that although ERK1 and ERK2 share 85% homology in amino acid sequence and are activated by the same factors and have the same substrates. ERK2 genetic knockdown is embryonic lethal, while ERK1 genetic knockdown causes impaired thymocyte maturation and synaptic plasticity [34], [35]. Additionally, studies have shown that ERK1 ablation in mouse embryo fibroblasts and NIH3T3 cells increases ERK2 dependent signaling and increases cell growth, whereas ERK2 knockdown decreases cell proliferation [36]. These differences in function between ERK1 and ERK2 mirror what we observe in our present study, where we find that ERK2 is more effective than ERK1 in mediating EMT, cell migration and cell adhesion. However, the discrepancies we observe between UO126 and ERK1 knockdown in MCF-7 Neo cells show that there is still more work that needs to be done to dissect out the role of ERK1. Our cell model was better able to dissect out the role of ERK2 on EMT in breast cancer.

Clinical trials have attempted to treat breast cancer with CI-1040 (PD184352), an orally active, highly potent selective MEK1 and MEK2 inhibitor, but were unsuccessful [22]. Breast cancer patients treated with epidermal growth factor receptor (EGFR) inhibitor, Gefitinib, suffered relapses, which were due to MAPK inhibition that paradoxically lead to PI3K/AKT activation [21]. Others have found that ERK2 also functions to promote therapy resistance [29], [37], [38]. Current clinical trials are testing the use of MEK and PI3K inhibitors in order to generate more effective therapeutic strategies. Our studies attempt to demonstrate that it may be possible to target breast cancer progression by targeting the ERK2 isoform, which will abrogate Snail-mediated signaling.

**Figure 7:**
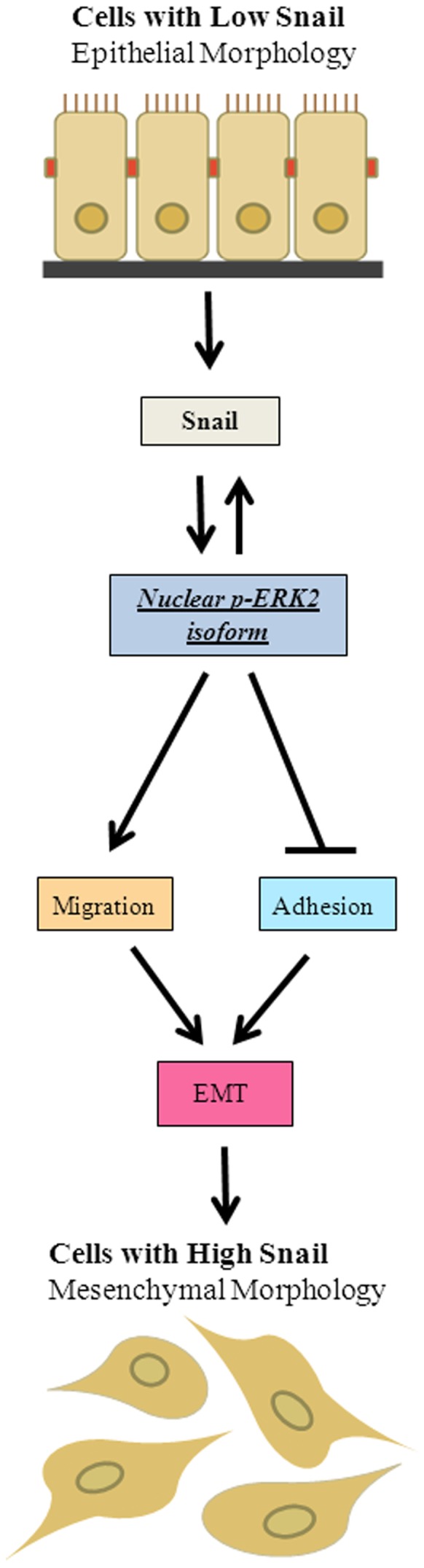
Snail promotes EMT via ERK2. Cells with low Snail maintain an epithelial morphology. During cancer progression, the expression of Snail increases, which promotes the nuclear localization of p-ERK2 isoform. This leads to EMT characterized by decreased E-cadherin, increased vimentin, decreased cell adhesion and increased cell migration. By a positive feedback loop, p-ERK can increase Snail expression. This leads to tumors with high levels of Snail and a mesenchymal morphology.

Collectively, our findings indicate for the first time that Snail-mediated induction of EMT, increased cell migration and decreased cell adhesion, may be mediated by p-ERK, and more specifically, p-ERK2 isoform (Figure **7**). This study demonstrates the significance of therapeutic targeting of Snail via targeting of the ERK2 isoform in future investigations on breast cancer.

## Supporting Information

Figure S1 Single and merged images for breast cancer tissue microarray double-labeled with p-ERK and Snail antibodies. (A) Normal breast tissue adjacent to cancer, (B) infiltrating ductal carcinoma grade 1, (C) infiltrating ductal carcinoma grade 2, (D) infiltrating ductal carcinoma grade 3. DAPI was used to stain the nuclei. Images were captured at 40× (oil) magnification.
(TIF)

Figure S2 Single and merged images from immunofluorescent staining for T47-D and MDA-MB-231 transiently transfected with Snail siRNA. T47-D and MDA-MB-231 transiently transfected with either control siRNA or Snail siRNA were analyzed by immunofluorescent staining for p-ERK, ERK and Snail. Snail (A, D), p-ERK (B, E), and ERK (C, F) primary antibodies were used to determine subcellular localization in samples. DAPI was used to stain the nuclei. Images were captured at 20× magnification.
(TIF)

Figure S3 Snail is expressed in mouse tumor xenografts overexpressing Snail. MCF-7 Neo and MCF-7 Snail cells were injected subcutaneously into female nude mice (N = 6) and 2 weeks later, mice sacrificed and tumor xenografts excised. Sections from the tumor xenografts were stained with (A) hematoxylin/eosin (H&E) to examine histology of the tissues as well as (B) Snail primary antibody by immunohistochemistry. Images were captured at 10× and 20× magnifications.
(TIF)

Figure S4 Snail and p-ERK co-localize in the nucleus of MCF-7 Snail transfectants while p-ERK is cytoplasmic in MCF-7 Neo cells. (A) Snail, (B), p-ERK (C) and ERK were analyzed by immunofluorescence in MCF-7 Neo and MCF-7 Snail cells. Images were captured at 20× magnification. (D) Another view of p-ERK in MCF-7 Neo cells is shown at 40× magnification. The cell membrane of one of the epithelial cells can be seen (white arrows) while the p-ERK is mostly cytoplasmic closer to the nucleus. DAPI was used to stain the nuclei.
(TIF)

Figure S5 Snail knockdown correlates with nucleo-cytoplasmic translocalization of p-Elk-1. MDA-MB-231 breast cancer cells were transfected with either control siRNA or Snail siRNA. Cells were analyzed by immunofluorescence with either (A) p-Elk-1 or (B) Elk-1 primary antibodies. DAPI was used to stain the nuclei. Images were captured at 20× magnification.
(TIF)
