# Supplementary figures and images for: Snail Promotes Epithelial Mesenchymal Transition in Breast Cancer Cells in Part via Activation of Nuclear ERK2
Source: PLoS One. 2014 Aug 14;9(8):e104987. doi: 10.1371/journal.pone.0104987 (PMC4133359; doi:10.1371/journal.pone.0104987)

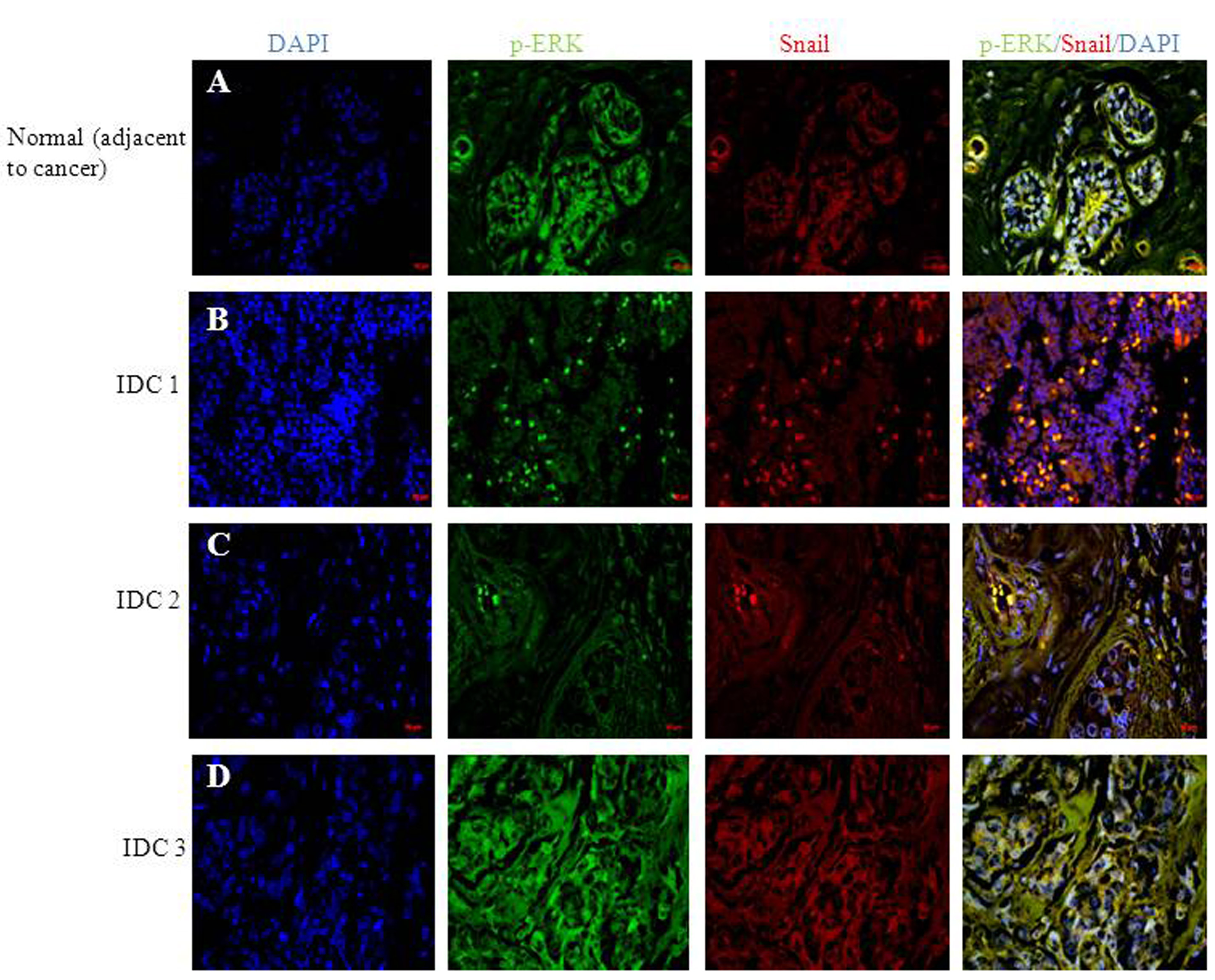

Supplement: Figure S1 — Single and merged images for breast cancer tissue microarray double-labeled with p-ERK and Snail antibodies. (A) Normal breast tissue adjacent to cancer, (B) infiltrating ductal carcinoma grade 1, (C) infiltrating ductal carcinoma grade 2, (D) infiltrating ductal carcinoma grade 3. DAPI was used to stain the nuclei. Images were captured at 40× (oil) magnification. (TIF) [file pone.0104987.s001.tif]

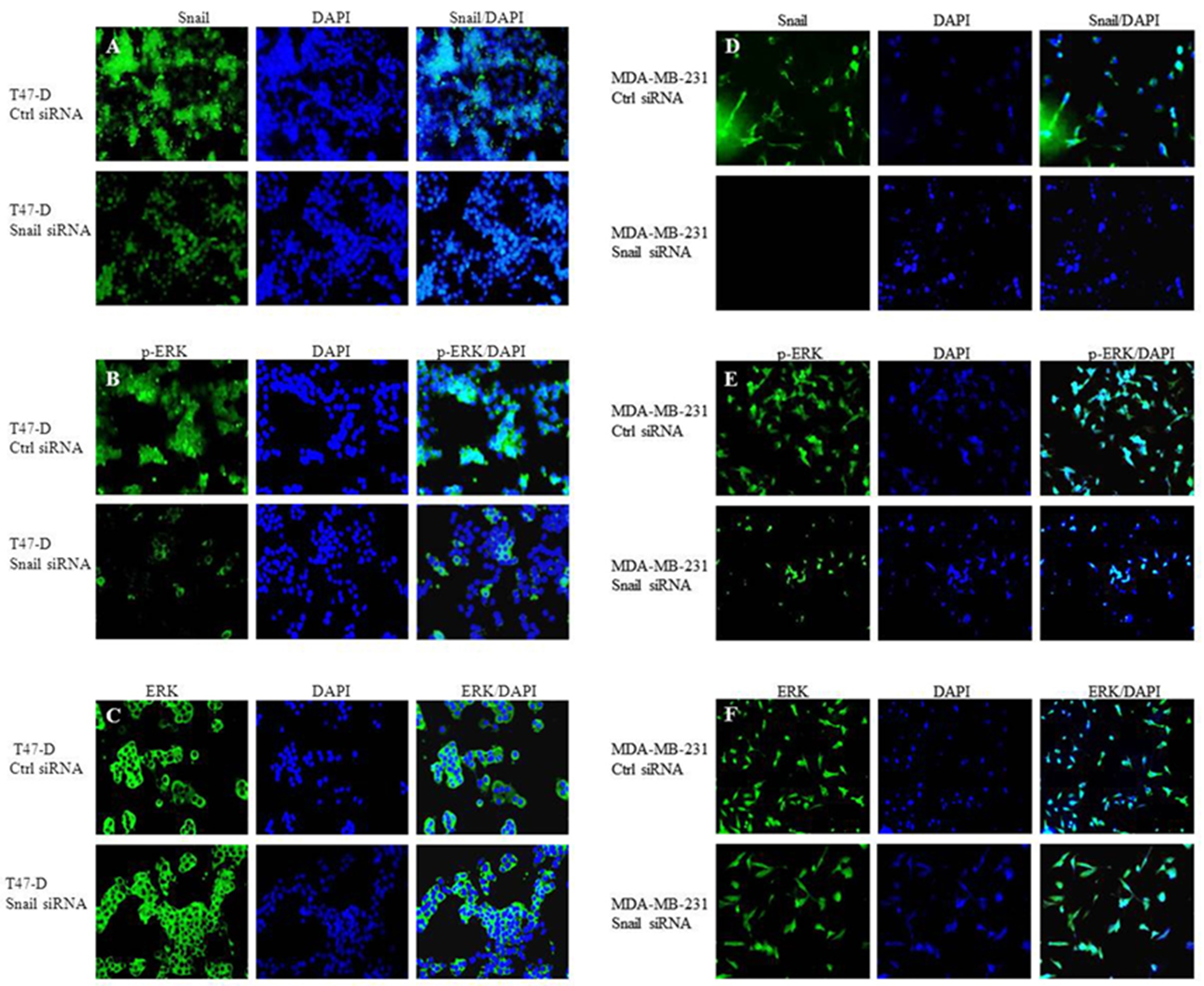

Supplement: Figure S2 — Single and merged images from immunofluorescent staining for T47-D and MDA-MB-231 transiently transfected with Snail siRNA. T47-D and MDA-MB-231 transiently transfected with either control siRNA or Snail siRNA were analyzed by immunofluorescent staining for p-ERK, ERK and Snail. Snail (A, D), p-ERK (B, E), and ERK (C, F) primary antibodies were used to determine subcellular localization in samples. DAPI was used to stain the nuclei. Images were captured at 20× magnification. (TIF) [file pone.0104987.s002.tif]

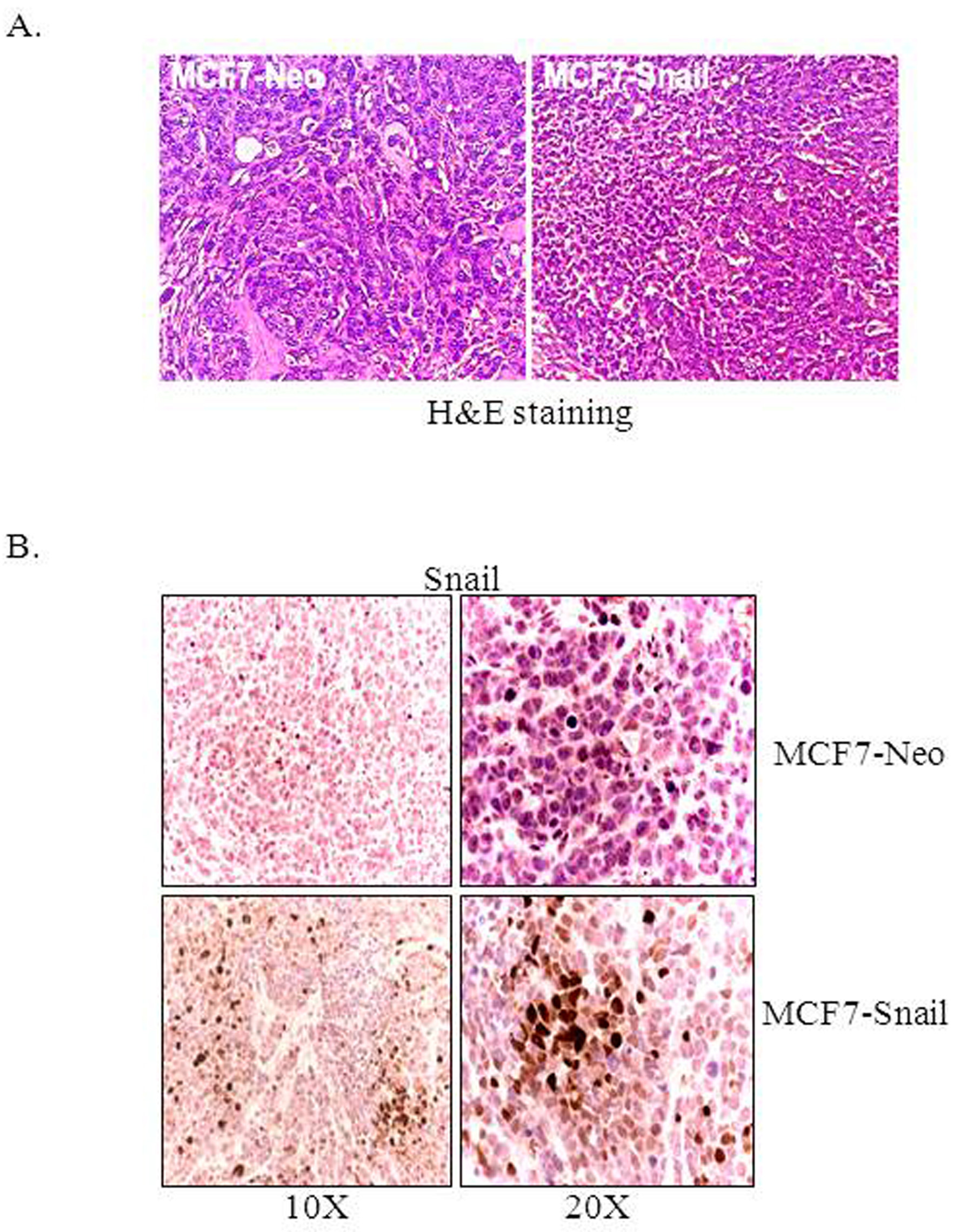

Supplement: Figure S3 — Snail is expressed in mouse tumor xenografts overexpressing Snail. MCF-7 Neo and MCF-7 Snail cells were injected subcutaneously into female nude mice (N = 6) and 2 weeks later, mice sacrificed and tumor xenografts excised. Sections from the tumor xenografts were stained with (A) hematoxylin/eosin (H&E) to examine histology of the tissues as well as (B) Snail primary antibody by immunohistochemistry. Images were captured at 10× and 20× magnifications. (TIF) [file pone.0104987.s003.tif]

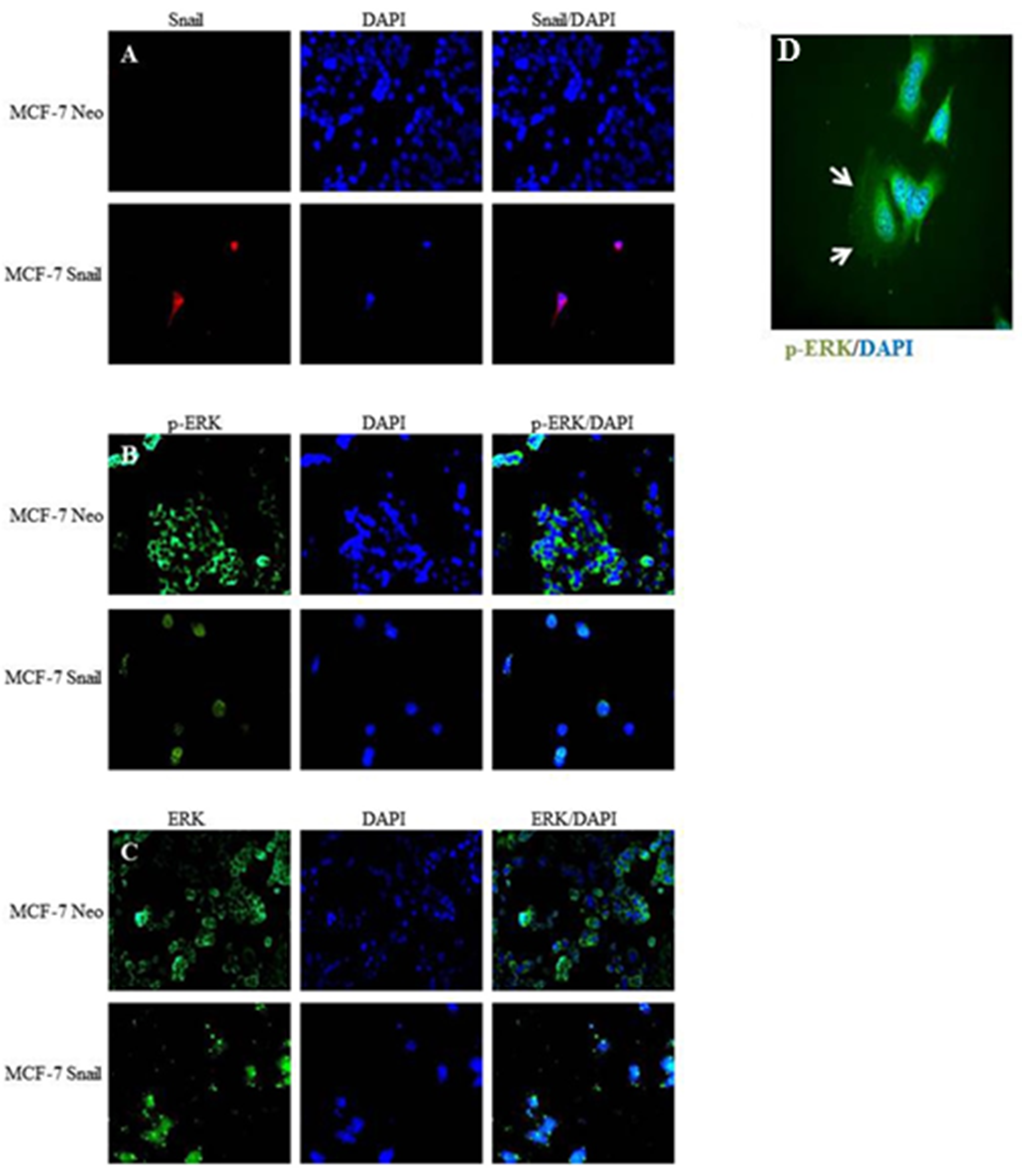

Supplement: Figure S4 — Snail and p-ERK co-localize in the nucleus of MCF-7 Snail transfectants while p-ERK is cytoplasmic in MCF-7 Neo cells. (A) Snail, (B), p-ERK (C) and ERK were analyzed by immunofluorescence in MCF-7 Neo and MCF-7 Snail cells. Images were captured at 20× magnification. (D) Another view of p-ERK in MCF-7 Neo cells is shown at 40× magnification. The cell membrane of one of the epithelial cells can be seen (white arrows) while the p-ERK is mostly cytoplasmic closer to the nucleus. DAPI was used to stain the nuclei. (TIF) [file pone.0104987.s004.tif]

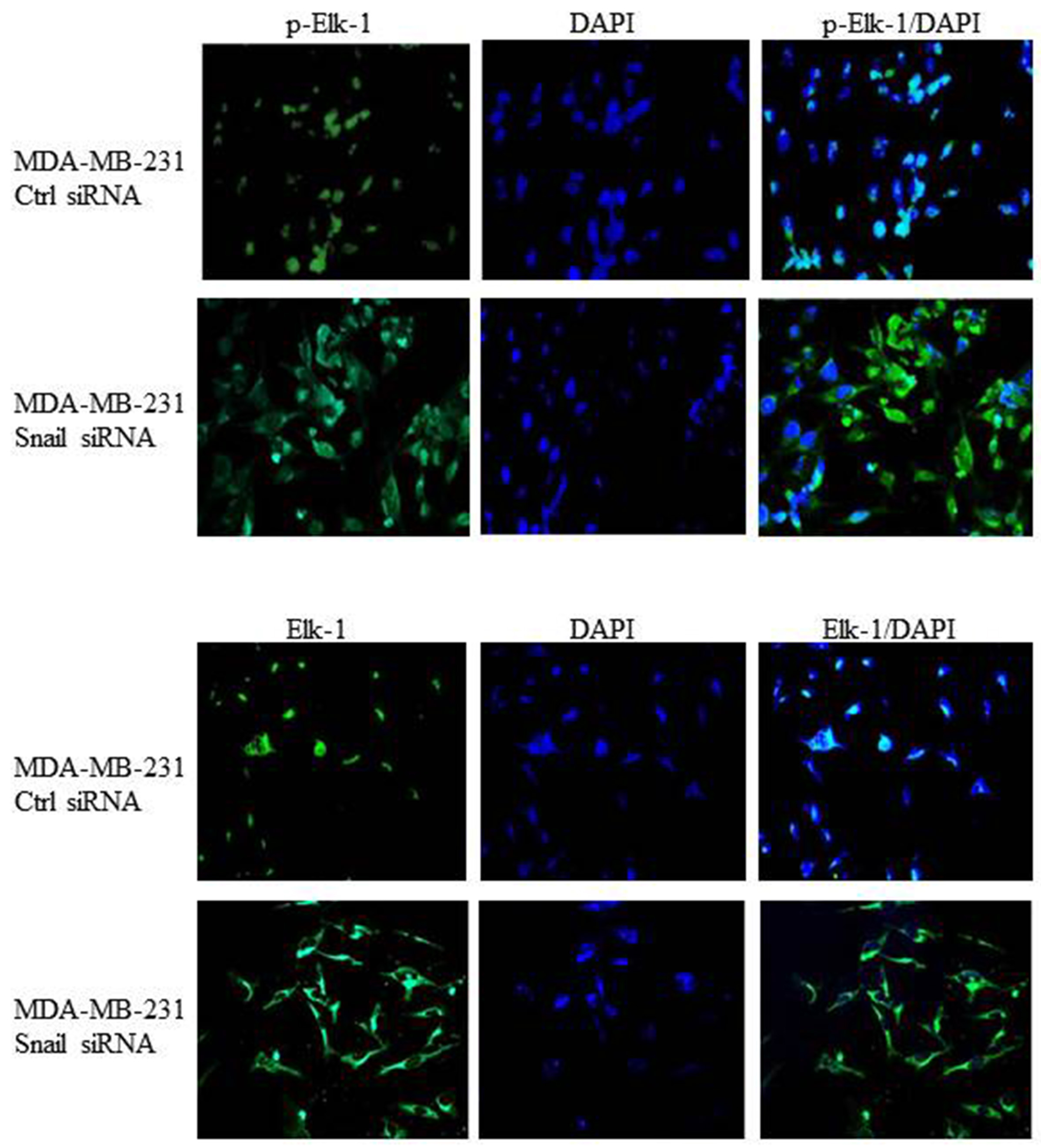

Supplement: Figure S5 — Snail knockdown correlates with nucleo-cytoplasmic translocalization of p-Elk-1. MDA-MB-231 breast cancer cells were transfected with either control siRNA or Snail siRNA. Cells were analyzed by immunofluorescence with either (A) p-Elk-1 or (B) Elk-1 primary antibodies. DAPI was used to stain the nuclei. Images were captured at 20× magnification. (TIF) [file pone.0104987.s005.tif]
